# HIV-infected patients in the ICU in the current era of high-activity antiretroviral treatment

**DOI:** 10.1186/cc9932

**Published:** 2011-03-11

**Authors:** P Vidal Cortés, V Aller Fernández, M Mourelo Fariña, P Lameiro Flores, P Vázquez Rodríguez, A Castro Iglesias

**Affiliations:** 1 CHU A Coruña, A Coruña, Spain

## Introduction

Our purpose is to study the effect of high-activity antiretroviral treatment (HAART) on the epidemiology and outcome of human inmunodefficiency virus (HIV) patients in the ICU. HAART has modified the outcome of patients infected with HIV, increasing survival and reducing infectious complications. In the first years of HAART use a significant change in the diagnosis and prognosis of ICU-admitted HIV patients has been identified, but there are no studies investigating this issue in the most recent years.

## Methods

A retrospective study. HIV patients admitted to a 36-bed ICU, between January 2005 and December 2009 (HIV incidence in our population: 42 cases/million hab/year). We studied demographic characteristics, having or not HAART, final diagnosis, need for organ support and outcome (length of stay (LOS) and mortality).

## Results

One hundred and five HIV-infected patients (70.5% being male), 52 (49.5%) having HAART. Mean age: 41 ± 8.57 years. More common co-morbidities were: hepatic disease (61%), cirrhosis in a 10.5%, followed by chronic respiratory disease and dyslipemia (12.4%), cardiac disease (5.7%), solid and hematologic malignancy (5.7% and 2.9%, respectively). A total 70.5% had a history of intravenous drugs use, and 13.3% were heavy alcohol consumers. Average CD4 count was 275.4 ± 362/ml, mean viral load was 3,656 ± 3,000/ml. A total 52.1% were on their CD4 nadir at admission time. Most frequent final diagnosis (grouped): infectious disease, 58.3% (focus: lung 66.7%, CNS 16.7%), cardiac disease (12.7%), intoxication and trauma (5.8% each one). Average APACHE II: 20.9. A total 48.6% of patients needed support with vasopressors, 64.7% mechanical ventilation and 15.2% renal support. A total 69.5% of patients needed at least one organ support. ICU LOS: 8.7 ± 9.9 days, hospital LOS: 29 ± 29.5. ICU mortality: 28.6%, hospital mortality: 35.2%.

## Conclusions

Despite the beneficial effects of HAART on inmune status, infection (especially pneumonia) remains the most common cause of ICU admission. Our results confirm the trend to a lower mortality saw in early HAART period studies.
